# Assessment of Performance of *Posidona oceanica* (L.) as Biosorbent for Crude Oil-Spill Cleanup in Seawater

**DOI:** 10.1155/2019/6029654

**Published:** 2019-10-31

**Authors:** Senda Ben Jmaa, Amjad Kallel

**Affiliations:** Laboratory of Water, Energy and Environment, Sfax National School of Engineering, University of Sfax, Sfax 3038, Tunisia

## Abstract

The marine environment is constantly at risk of pollution by hydrocarbon spills that requires its cleanup to protect the environment and human health. *Posidonia oceanica* (L.) (PO) beach balls, which are characteristic of the Mediterranean Sea and abundant on the beaches, are used as biosorbent to remove hydrocarbons from the sea. The impact of several factors such as oil concentration, time sorption, and weight sorbent was investigated to determine the oil and water sorption capacity for raw and milled *P. oceanica* fibers. The study of kinetic models for initial crude oil concentration of 2.5, 5, 8.8, 10, 15, 20, 30, and 40 g/L revealed that crude uptake followed the pseudo-first-order model while, for isotherm models, the crude uptake onto the *P. oceanica* tended to fit the Langmuir model. Experiments were performed according to two systems: a pure oil and pure water system and a mixed oil/water system. For the dry system (pure oil and pure water), the maximum oil and water sorption capacity of raw and milled fibers was found to be 5.5 g/g and 14 g/g for oil and 14.95 g/g and 15.84 g/g for water, respectively, whereas, in the mixed oil/water system, the maximum oil and water sorption capacity was estimated as 4.74 g/g, 12.80 g/g and 7.41 g/g, 8.31 g/g, respectively. The results showed that, in spite of their absorbency of a lot of water, the milled fibers with grain size ranging between 0.5 mm and 1 mm might be the relevant sorbent for the elimination of crude oil from seawater thanks to its efficient sorption capacity and low cost.

## 1. Introduction

Oil remains one of the main resources of energy in the modern industrial sector as well as an important source of raw materials for synthetic polymers and chemicals worldwide [1]. The environment, however, has borne the brunt of oil exploration and transportation for several decades resulting in oil spills responsible for contamination of coastal waters and land. Oil spills regrettably occur very often due to the escape of oil from crude oil confinements, offshore platforms and drilling rigs, and spills of refined petroleum products and their by-products. These spills harm aquatic life like birds and shellfishes, and the environment like shorelines, mosses, and sea creatures. This has an adverse effect on the economy, disrupting mainly the recreation and tourism activities. Weathering, technically speaking, is the process, which includes, respectively, the spreading, evaporation, dispersion, dissolution, oxidation, emulsification, sedimentation, and finally biodegradation of oil spilled into the sea. Any spill-treatment method depends largely on these processes [2]. Given the oil impacts on the environment, it is important to prioritize actions to improve response to a spill and, thus, protect the environment. The cleanup of an oil spill can be carried out by three groups of technologies, namely, biological methods; mechanical/physical recovery methods, such as booms, skimmers, and adsorbents; and chemical techniques such as dispersion, in situ burning, and solidifiers. The main limitations of the mechanical and chemical treatment methods are their inefficient trace level adsorption and their high costs [2]. Thus, sorption is the most favored procedure for cleaning oil-spill because of its low cost. Besides, it is highly efficient and more environmentally friendly. Oil can be removed from water by sorbents without the risk of draining out (i.e., of oil). Indeed, sorbents are able to pull together liquids and transform them into the semisolid or solid state. Hence, when an oil sorbent is used in the cleanup process, no secondary pollution is produced. Furthermore, the operation process itself is easy and the costs remain comparatively low [3].

In recent years, the sorption of oil by bioadsorbents has been found as an excellent alternative to mitigate the contamination and remedy the places of the spill [4]. Natural organic materials and composite materials have been applied to clean up oil spills. Several researchers have developed a number of natural organic materials as an adsorbent material, such as barley straw [5], rice straw [6], rice husk [7], pith bagasse [8], banana peels [2], Kapok Fiber [9], Sawdust [10], garlic and onion peel [11], palm empty fruit brunch and leaves [12], and cotton [13].

Unlike other types of sorbents, organic sorbents are suitable resources used for sorption oil since they are readily accessible and relatively cheap. Moreover, they have the advantage of being fully biodegradable and environmentally friendly, leaving no harmful residues [12]. *Posidonia oceanica* (L.) (PO) is an abundant sea grass of the Mediterranean basin, whose remains of old leaf sheaths on the seashore are massively accumulated as fibrous dense balls made of cellulose and hemicellulose (about 60–75%) and lignin (about 25–30%) [14, 15]. Although considered as aesthetically annoying, recent studies have focused on a variety of potential uses of such biowaste. Allouche et al. [14] used PO to adsorb Pb (II) and obtained significant results. Wahab et al. [16] employed PO to remove and recover phosphorus from aqueous solutions. Jellali et al. [17] also used PO to adsorb ammonium from aqueous solutions. Ncibi et al. [18] studied the adsorption of methylene blue onto PO. The sorption of yellow 59 onto PO was studied by Guezguez et al. [19]. In addition, the biosorption of 2,4-dichlorophenol (2,4-DCP) onto PO seagrass was studied by Demirak et al. [20] in batch system. All of these studies show that natural PO is an effective absorbing material and that it could have potential uses in oil-spill cleaning.

The aim of this study is to evaluate the ability of low-cost PO raw fibers, which is a readily available organic material (collected from beaches as solid waste), to clean up oil spills while taking into consideration various factors. Batch experiments are conducted in two systems: dry system (seawater or oil) and oil/water layers system. The dynamics/performance of the sorption process and the effect of variable parameters such as sorbent weight, sorption time, particle size, and oil concentration are investigated.

## 2. Materials and Methods

### 2.1. Biosorbent Preparation and Characterization

**Figure 1:**
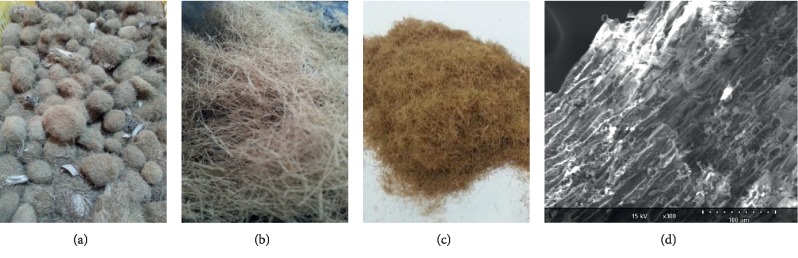
*Posidonia oceanica* (PO) used as biosorbent: (a) collected balls, (b) separated raw fibers, (c) milled and sieved PO fibers, and (d) SEM of PO fibers.

The beach's balls of PO (Figure 1(a)) were collected from Chaffar beach (Tunisia) as solid waste. Their fibers were manually separated (Figure 1(b)). Distilled water was used in the cleaning process to remove salt and other impurities from the PO fibers. After that, the obtained fibers were oven-dried at 40°C for 72 hours until their weight become constant. The dried-up fibers were then put in storage in desiccators. The material obtained at this stage was either used in its raw state or turned into particles with a Willy mill and segregated (with a sieve) according to their sizes which ranged from 0.063 mm to 2 mm (Figure 1(c)). The fibers of PO were subject to Fourier Transform Infrared (FTIR) spectroscopy and biochemical and elemental contents analysis.

100 mg of dried KBr was mixed with 1 mg of each sample to be used in spectroscopic measurements. The FTIR spectra are ranged from 500 to 4000 cm^−1^. A Nicolet FTIR spectrometer was used to detect the important peaks located in the sample. The data for pure KBr was used to correct the background noise.

The Kjeldahl method was used to determine the content of protein in PO fibers according to AOAC methods number 984.13 [21]. The phenol-sulfuric acid was used to estimate the total carbohydrates and measurement of the absorbance was done at 490 nm [22]. The content of the ash was evaluated by maintaining the sample in a muffle furnace heating for 2 h at 650°C in the presence of air, until a constant mass was reached [23]. 2 g of dried fibers was heated in an oven at 105°C until the dried matter became constant. The difference of weight gives the moisture content [24].

The elemental content (Ca, Mg, K, Fe, Na, Zn, and Mn) was determined by flame atomic absorption spectroscopy (AAS) on acid digested and heated ash.

### 2.2. Batch Experiments for Sorption Capacity

The sorption experiments were conducted in batch for two different systems: dry system (oil only and water only) and oil/water layer for which sorption kinetics/equilibrium and the effect of milling/particle size were investigated. The oil was a crude obtained from a local oilfield and used as received (density 0.866 g/cm^3^ and mole weight 221–225 g/mol). The water used was seawater (pH 7.45 and salinity 42.89 g/L) brought from the same area as the PO balls.

#### 2.2.1. Sorption Capacity in Single Oil and Water Systems

An amount of dried PO sample was put into a beaker containing 30 ml of crude oil or water at room temperature and stirred moderately for various periods of time. The sample was filtered through a mesh that had already been weighed. The sorbent was then drained and the excess oil or water was wiped from the mesh with filter paper. The determination of the oil sorption capacity *Q*_O_ or the water sorption capacity *Q*_W_ (g oil or water per g of sorbent) was carried out by weighing the samples before and after the sorption.

#### 2.2.2. Sorption Capacity in Oil/Water System

Different amounts of crude oil were added to 50 mL of seawater in a 100 mL flask and shaken for 10 min to mimic the effect of the waves in pushing the layer of oil up to the surface of the sea. After that, various quantities of PO were spread over the oil-water mixture and shaken for a few minutes (up to the investigated reaction time) at 25°C. After that, the soaked PO fibers were removed from the flask using mesh screen, drained for 5 min, and weighed. The mesh screen was then washed with *n*-hexane to remove any oil and/or water stacked on it, back into the experiment flask. The total of the remaining content of oil was determined according to the EPA 1664 method [25]. Thereafter, the remaining oil was subtracted from the initial amount and the sorption capacity was evaluated by the following equation (1):

(1) $$\begin{array}{c} q=\frac{W_{\text{i}}-W_{\text{r}}}{W_{\text{P}}},\; \end{array}$$

where *q* is the capacity sorption of oil of the sorbents (gram of oil per gram of sorbent), *W*_i_ (g) is the initial mass of the oil used in the oil-water system, *W*_r_ (g) is the remaining (not sorbed) amount of oil, and W_P_ (g) is the weight of PO used in the process.

The water sorption capacity in the oil/water system (gram of water per gram of sorbent) was inferred by subtracting the *W*_P_ and the weight of sorbed oil from the weight of soaked/drained PO fibers divided by *W*_P_.

## 3. Results and Discussion

### 3.1. Characterization of the Biosorbent

The biochemical composition, ash and moisture content, and the elemental analysis of dry PO fibers are illustrated in Table 1.

The biochemical composition shows that total carbohydrates constitute the major components at 79.7% and the contents of ash and moisture are 7.3% and 10.9%, respectively. The elemental analysis reveals that sea grass fibers are rich in cations. The results have shown that calcium (97.3 mg/g), magnesium (42.3 mg/g), sodium (38.7 mg/g), iron (33.3 mg/g), and potassium (10.7 mg/g) are the main cations in PO, as determined by flame atomic absorption spectroscopy (AAS).

The biosorbents had a considerable number of functional groups on its surface which were determined by using the FTIR spectroscopy.

**Figure 2:**
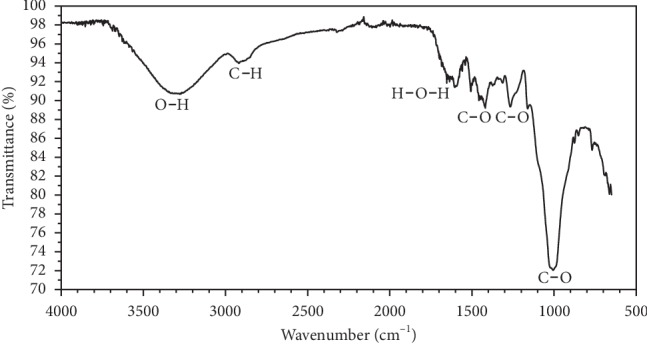
FTIR spectra of raw PO fibers.

The spectra of raw PO shown in Figure 2 display several adsorption peaks, indicating that the biomass has a complex nature. The vibrations stretching of the hydroxyl groups (O-H) presented in lignin, hemicellulose, and cellulose of PO fibers were detected at 3300–3400 cm^−1^ peaks. The symmetric –CH_2_ valence vibration and –CH stretching vibration were shown at 2920 cm^−1^ and 2860 cm^−1^ peaks. Absorbed water (H-O-H) and C-O stretching vibration in ester were revealed at 1636 cm^−1^ and 1267 cm^−1^ peaks [2]. The most important peak is the one observed at 1006 cm^−1^ which corresponds to C–O stretching being associated with the presence of cellulose, hemicellulose, and lignin [26]. Linkages between the sugar units in celluloses and hemicelluloses by a *β*-glycosidic bond were seen at 888 cm^−1^ peaks [27].

The high quantity of calcium cations was confirmed with the presence of a peak at 1377 cm^−1^ that corresponds to a pectin peak. The calcium bridges have been built between two carboxyl groups of two adjacent molecules of pectin by these calcium cations [28].

### 3.2. Oil and Water Sorption Capacity of Raw and Milled Fibers in Dry Systems

Preliminary tests were conducted in dry systems (single water and oil systems) to find out optimum operating conditions and parameters, such as sorbent concentration, reaction time, and grain size.

The interaction between the oil and the sorbent could be affected by the sorbent dosage. It has been found that increasing the sorbent doses decreases the sorption capacity. In fact, the decrease of the adsorption capacity with the sorbent amount is due to the higher unsaturated adsorption sites that remain after the adsorption reaction which is similar to the trend observed by Abdelwahab [29] for diesel and heavy crude oil adsorption onto raw luffa, whereas Husseien et al. [5] reported the opposite when they studied the absorbance of Marine Balaiem crude oil by barley straw.

The milled fibers were classified in different fraction sizes and each one of them was tested for 5, 10, and 15 min reaction time. The results presented in Table 2 reveal an optimum of oil sorption obtained with the size fraction ranging from 0.5 to 1 mm where the sorption capacity is up to 14 g/g which is about threefold the sorption obtained with raw fiber. This increase in the oil sorption capacity after milling is due mainly to the increase in surface area. On the other hand, particles finer than 0.5 mm showed a lower sorption capacity. This is probably due to the fact that the pores and capillaries present between the fibers become plugged when small particles (i.e., <0.5 mm) stick together [2].

**Figure 3:**
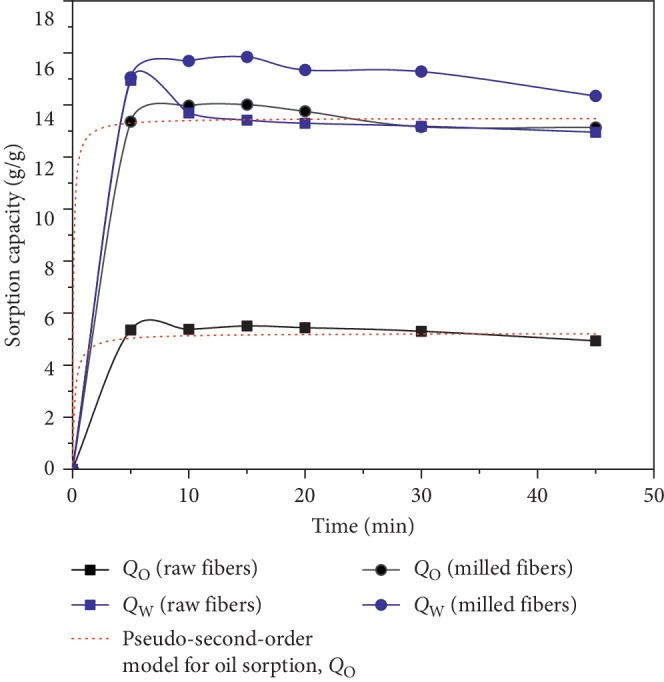
Oil and water sorption capacity in single system (*Q*_O_ and *Q*_W_) at various reaction times (with 0.1 g of PO fibers).

The oil and water sorption capacity in single system (*Q*_O_ and *Q*_W_) of raw and milled PO fibers (0.5 mm-1 mm), which was investigated at different times 5, 10, 15, 20, 30, and 45 min, is displayed in Figure 3.

The capacity sorption of the sorbents (raw and milled fibers) rapidly increased with time, up to about 15 min when the sorption process reached equilibrium. The maximum oil and water sorption capacity for raw and milled fiber was 5.5 and 14 g/g for oil and 14.9 g/g and 15.8 g/g for water, respectively. Further increase of time slows down the process due to the availability of a limited number of sites for the crude oil to get adsorbed [30], especially for water sorption on raw fibers where a light desorption was noticed beyond 5 min of contact time. It has been shown that raw fibers of PO have the ability to adsorb more water rather than oil. In fact, PO fibers are rich with hydrophilic groups (COOH, OH, and others) found in cellulose and hemicellulose which are capable of attracting water. Such affinity is common for natural fibers, which tend to absorb water and get swollen [31]. Djati Utomo et al. [32] reported a similar water sorption of about 15 g/g for sugarcane bagasse. Even though a similar trend has been detected for milled fibers, the increase of specific surface has drastically enhanced the oil sorption capacity.

### 3.3. Study of Sorption in Oil/Water System

The sorption of crude (mixed in water) onto PO milled fibers was investigated for the effect of sorbent weight, reaction time, and crude initial concentration, and the dynamics of the process (kinetics and isotherms) was modeled.

#### 3.3.1. Effect of Sorbent Dose

**Figure 4:**
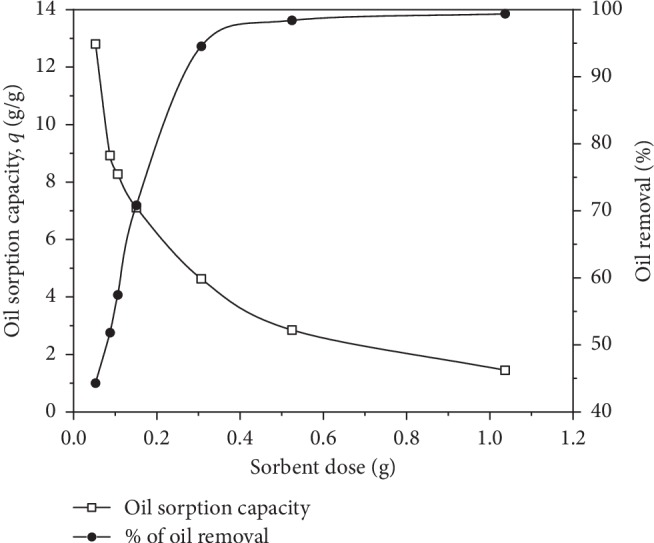
Effect of biosorbent dose on oil sorption capacity and oil removal efficiency (reaction time = 15 min, oil concentration = 30 g/L).

The impact of sorbent dose on the amount of crude oil removal was studied for different sorbent doses ranging from 0.05 to 1 g (i.e., 1 to 20 g/L) at 30 g/L of crude in seawater and the findings are presented in Figure 4. The efficiency of oil removal is linearly increased from 45% to 95% when the PO weight was augmented from 1 to 6 g/L. This rise is attributed mainly to the availability of more adsorption sites and the augmentation in the sportive surface area [18]. At higher sorbent doses, 10 and 20 g/L, the removal of oil up to 98.5% and 99.5%, respectively, was achieved. On the other hand, the oil sorption capacity per unit mass of PO considerably decreased, basically due to the higher unsaturated sites and remaining unoccupied surface during the process. Indeed, the active surface increased for the same concentration of crude resulting in a decreased per unit weight PO value [33].

#### 3.3.2. Kinetics Study

**Figure 5:**
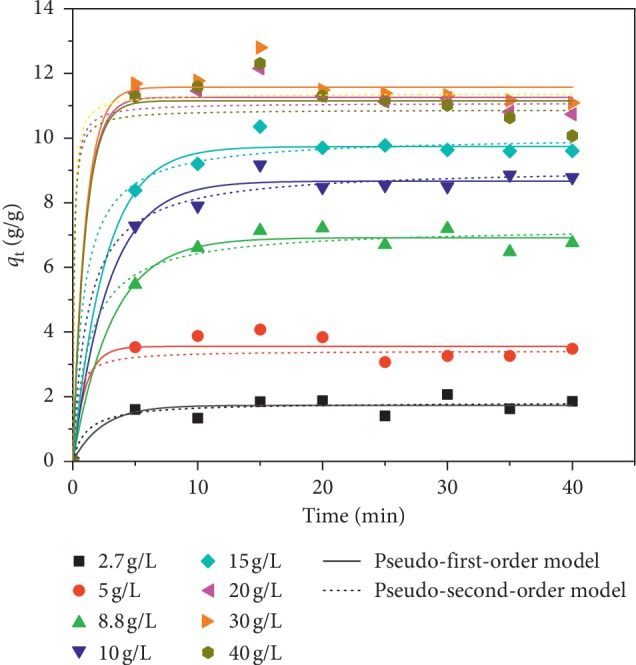
Sorption kinetics of oil onto PO milled fibers for different initial concentration of crude (biosorbent dose = 0.05 g; solid lines and dotted lines represent the fitting curves with pseudo-first-order and pseudo-second-order models, respectively).

The results of oil sorption tests in an oil/water system with 1 g/L biosorbent (milled fraction) and changing the reaction time (0–40 minutes) and the initial concentration of oil *C*_0_ (2.5, 5, 8.8, 10, 15, 20, 30, and 40 g/L) are depicted in Figure 5.

Our findings show the existence of at least three states in each curve. The first one, which is the initial stage in the sorption process, takes place during the first 5 minutes. This period is characterized by its high sorption rate. The transitional state, during which sorption reaches its maximum, occurs at 15 min. Beyond 15 min, the amount of crude absorbed by the sorbent is barely affected, making this state a relatively steady-state period.

The adsorption process rate and its effect on the equilibrium time are often determined by kinetic models. The mass transport process and chemical and/or physical characteristics of the adsorbent affect the adsorption mechanism [34]. In order to explore the mechanism of biosorption and potential rate controlling steps, such as chemical reaction processes and mass transport, the distinguished pseudo-first-order, pseudo-second-order models have been applied to evaluate the experimental data.

Nonlinear forms of the pseudo-first-order and the pseudo-second-order models are given by equations (2) and (3), respectively [35]:

(2) $$\begin{array}{c} q_{\text{t}}=q_{\text{e}}\left(1-e^{-k_{1}t}\right), \end{array}$$

(3) $$\begin{array}{c} q_{\text{t}}=\frac{k_{2}q_{\text{e}}^{2}t}{1+k_{2}q_{\text{e}}t}, \end{array}$$

where *q*_t_ and *q*_e_ are the quantity of adsorbed oil (g/g) at time *t* and at equilibrium, respectively, and *k*_1_ (1/min) and *k*_2_ (g/g min) are the equilibrium rate constant of first order and second order, respectively. The experimental data for each initial concentration were fitted to the models' equation by means of an iterative nonlinear technique, using Microcal Origin Software to determine the parameters' values for each model. The best fit was satisfied according to the following three conditions: (i) the *q*_e_ values should reasonably match with the experimental amount adsorbed when equilibrium has been reached, (ii) the values of *R*^2^ should be maximum, and (iii) the values of *χ*^2^ should be minimum. The fitting parameters derived for both models are shown in Table 3 while the fitting curves are presented in Figure 5.

All curves appear to stabilize after the first 10 minutes where most of the crude was adsorbed within five minutes. As can be seen in the figures, the variation noticed after the equilibrium phase might reflect a slight sorption or desorption after the saturation process. Such behavior was also revealed by Brandão et al. [36] and Almeida et al. [1].

Both models exhibited a good fitting to the experimental data with slightly better results for the pseudo-first-order model, as can be seen by the values of *R*^2^ and *χ*^2^. The pseudo-second-order yielded higher rate constants (*k*_2_ almost twofold that of *k*_1_) which predict a fast process, but without evaluating the transient period (from 0 to 5 min), it is difficult to confirm.

#### 3.3.3. Sorption Isotherm

Adsorption isotherms (i.e., adsorption properties and equilibrium data) define the interaction between pollutants and adsorbent materials. Therefore, they are crucial in the optimization of sorbent use. Indeed, it is primordial to precisely quantify the amount of oil on the water surface in order to use any kind of sorbent effectively in practical oil-spill cleanup [37].

When the sorption reaction reaches equilibrium, the distribution of the solute between the liquid and solid phases is said to be balanced. The sorption isotherm is defined as the amount of solute sorbed relative to the amount of solute in solution at equilibrium [38]. To provide insight into sorption properties and equilibrium data of the current study, the Langmuir, Freundlich, Redlich-Peterson, and Jovanovic isotherms are used. The sorption process is then discussed in terms of constants that are characteristic of the individual systems. Each of these models has its own assumption and expression:

- The Langmuir model is defined as a homogeneous surface adsorption process. It assumes a monolayer coverage of the adsorbent without any interactions between sorbed molecules [39]. It is expressed by equation (4):

(4) $$\begin{array}{c} q_{\text{e}}=\;\frac{q_{\text{m}}K_{\text{L}}C_{\text{e}}}{1+K_{\text{L}}C_{\text{e}}}, \end{array}$$

where *q*_e_ (g/g) is the quantity of adsorbed oil at the time of equilibrium or monolayer adsorption, *q*_m_ is the maximum adsorption capacity (gram oil per gram sorbent), *C*_e_ (g/L) is the equilibrium concentration of the oil, and *K*_L_ is the Langmuir constant related to the adsorption rate.

(ii) Freundlich's isotherm (equation (5)) is determined by an empirical equation, which describes a multilayer adsorption with interaction between the adsorbed molecules. This process of adsorption is used for heterogeneous surfaces [39].

(5) $$\begin{array}{c} q_{\text{e}}=K_{\text{f}}C_{\text{e}}^{1/n}, \end{array}$$

where *K*_f_ and *n* are the Freundlich constant and intensity, respectively.

(iii) The isotherm of Redlich-Peterson is a mix of the Freundlich and Langmuir isotherms incorporating features from both models into the following expression (equation (6)) [40]:

(6) $$\begin{array}{c} q_{\text{e}}=\;\frac{K_{\text{R}}C_{\text{e}}}{1+A_{\text{R}}C_{\text{e}}^{\beta }}, \end{array}$$

where *K*_R_ and *A*_R_ are Redlich-Peterson constants and *β* is an exponent that lies between 0 and 1.

(iv) The Jovanovic isotherm is a model similar to the Langmuir one in terms of Localized monolayer adsorption, but it is characterized by lateral interactions between adsorbed molecules [41]. This model considers the former for the surface binding vibrations of an adsorbed species. The following nonlinear relationship (equation (7)) defines Jovanovic's isotherm:

(7) $$\begin{array}{c} q_{\text{e}}=q_{\text{m}}\left(1-e^{-K_{\text{J}}C_{\text{e}}}\right), \end{array}$$

where *K*_J_ is the Jovanovic isotherm constant (L/g), and *q*_m_ is the maximum sorption capacity in Jovanovic model (g/g).

**Figure 6:**
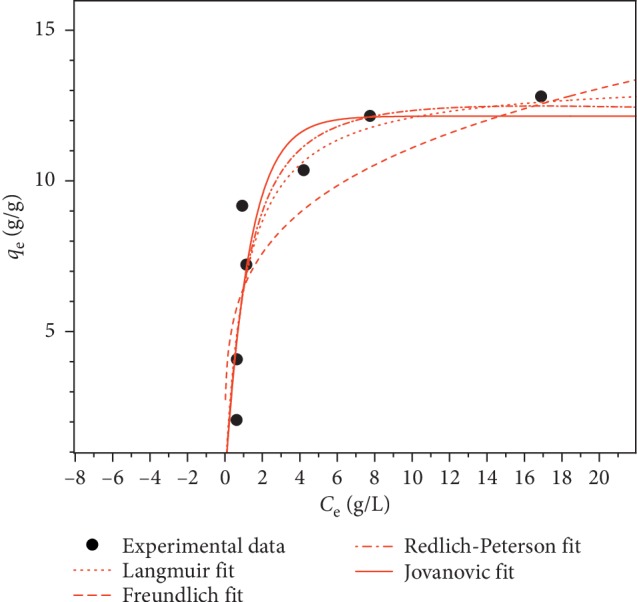
Sorption isotherms of oil onto PO milled fibers and fittings with Langmuir, Freundlich, Redlich-Peterson, and Jovanovic models (biosorbent dose = 0.05 g).

Figure 6 illustrates the relationship between oil concentration and the sorption capacity at equilibrium, as well as the best fit for the three isotherm models. The calculated parameters for each model are presented in Table 4.

Even though Freundlich isotherm's constant (*n*) ranged between 0 and 10, indicating a promising adsorption, this model exhibited the poorest fit among the studied models. The model of Langmuir fitted better the experimental data for which a maximum sorption capacity of 13.43 g/g was found. A dimensionless, constant, equilibrium parameters, or separation factor *R*_L_ (equation (8)) is the important characteristics of the Langmuir isotherm [40, 41].

(8) $$\begin{array}{c} R_{\text{L}}=\;\frac{1}{1+K_{\text{L}}C_{0}}, \end{array}$$

where *C*_0_ is the initial concentration (g/L) and *K*_L_ is the Langmuir constant.

The unfavorable adsorption is obtained when the value of *R*_L_ is bigger than 1. The linear adsorption is observed with *R*_L_ values equal to one. The favorable adsorption is revealed when *R*_L_ values are between 0 and 1. Zero *R*_L_ values indicate an irreversible adsorption [42]. For all of the initial concentrations investigated in this research, *R*_L_ values range between 0.03 and 0.3, which indicates that sorption is highly favorable.

The Redlich-Peterson model gave the best fit for the sorption process. Indeed, the value of *β* (1.078) is closer to unity than 0, which means that the isotherm is closer to the Langmuir than to the Freundlich model [43]. Furthermore, the Jovanovic model also showed relatively good fitting results (*R*^2^ = 0.899 and *χ*^2^ = 2.583) and confirms the probability of some mechanical contacts between the adsorbate and adsorbent [44]. Hence, the good fitting results with Langmuir, Redlich-Peterson, and Jovanovic isotherm expressions confirm the monolayer coverage process of crude onto homogeneous sites of PO fibers rather than a multilayer sorption process on heterogeneous surfaces.

The oil sorption efficiency of PO is compared to the results of various agricultural wastes/biomass reported in previous studies in terms of sorption capacity, as listed in Table 5. The sorption capacity of PO was significantly higher (up to 13.5 g/g) compared to several natural sorbents such as bagasse [48], wheat straw [47], and barley straw [5]. Other experimented materials have shown higher oil sorption than PO such as cotton fibers [13], silkworm cocoon waste [45], and silk-floss [46]. It is worth mentioning that the facts that PO fibers are readily available—which can be collected from beaches as solid wastes—make it a promising cost-effective material for oil-spill cleanup. Furthermore, the crude soaked onto natural PO could be easily recovered and the used fibers might offer a valuable high calorific source of energy.

## 4. Conclusions

The performance of *Posidonia oceanica* was studied to determine its capability for oil-spill cleanup. In this study, raw and milled fibers of PO were tested for their oil and water sorption capacity and the results revealed an oil sorption capacity up to 15.8 g/g with milled PO in a dry system. The study of the sorption process dynamics and equilibrium in oil-water system proved that 1 g of PO could achieve an uptake up to 13 g of oil from seawater, within few minutes. Concerning the isotherms and kinetics, Langmuir and pseudo-first-order models gave the best description of the nature of crude sorption onto *P. oceanica*.

It can be stated that PO fibers constitute a cost-effective sorbent for oil-spill cleanup. Additional merits, i.e., recycling, recovered oil and high calorific value of soaked fibers, support the use of PO as a promising and efficient oil cleanup material. Nevertheless, further investigation on possible chemical and/or thermal treatment of PO fibers could enhance its oil sorption performance.

## Figures and Tables

**Table 1:** Biochemical composition, ash and moisture content, and elemental analysis of raw PO fibers.

	Content (fresh basis)
*Biochemical composition*	*(%)*
Total carbohydrates	79.74
Protein	237
Fat	0.42
Nitrogen	0.38
Ash	7.25
Moisture	10.91

*Elemental analysis*	*(mg/g)*
Calcium	97.27
Magnesium	42.31
Sodium	38.69
Iron	33.25
Potassium	10.74
Zinc	0.75
Manganese	0.21

**Table 2:** Effect of particle size on oil sorption capacity in dry system (with 0.1 g of PO fibers).

PO fraction size (mm)	Oil sorption capacity, *Q*_O_ (g/g)		
	5 min	10 min	15 min
0.063–0.1	8.36	9.99	9.82
0.1-0.2	10.59	10.33	10.44
0.2–0.5	11.22	11.80	12.11
0.5–1	13.36	13.97	14.01
1–1.6	12.68	12.69	13.26
1.6–2	12.05	12.70	13.62
>2	10.72	11.95	11.82
Raw fibers	5.35	5.38	5.50

**Table 3:** Pseudo-first-order and pseudo-second-order kinetics parameters at different crude initial concentrations.

*C*_*0*_ (g/l)	Pseudo-first-order					Pseudo-second-order		
	*q*_e_ (g/g)	*k*_1_ (min^−1^)	*χ* ^*2*^	*R* ^*2*^	*q*_e_ (g/g)	*k*_2_ (g/g min)	*χ* ^*2*^	*R* ^*2*^
2.7	1.731	0.097	0.061	0.839	1.823	0.153	0.055	0.853
5	3.554	0.133	0.123	0.918	3.421	0.253	0.186	0.877
8.8	6.916	0.111	0.074	0.986	7.251	0.242	0.133	0.975
10	8.669	0.128	0.101	0.988	9.090	0.210	0.102	0.988
15	9.736	0.115	0.083	0.992	10.070	0.224	0.127	0.988
20	11.260	0.168	0.197	0.986	11.082	0.309	0.294	0.979
30	11.574	0.206	0.297	0.980	11.380	0.362	0.404	0.973
40	11.152	0.250	0.435	0.969	10.880	0.444	0.608	0.957

**Table 4:** Isotherms constants of fitting experimental data with Langmui, Freundlich, Redlich-Peterson, and Jovanovic models.

*Langmuir model*	
*q*_m (g/g)_	13.432
*K*_L_	0.909
*R* ^2^	0.835
*χ* ^2^	3.083

*Freundlich model*	
*K*_f_	6.453
*n*	4.248
*R* ^2^	0.710
*χ* ^2^	5.419

*Redlich-Peterson model*	
*K*_R_	10.339
*A*_R_	0.617
*β*	1.078
*R* ^2^	0.902
*χ* ^2^	2.949

*Jovanovic model*	
*q*_m (g/g)_	12.148
*K*_J_	0.767
*R* ^2^	0.899
*χ* ^2^	2.583

**Table 5:** Comparison of oil sorption capacities from this study and other oil-sorbing fibers.

Sorbing material	Type of oil	Sorption capacity (g/g)	Reference
Raw cotton fiber	Crude oil	30.5	[13]

Raw kapok fiber	Diesel	38.1	[37]
	Soybean oil	49.1	

Silkworm cocoon waste	Motor oil	42–52	[45]
	Vegetable oil	37–60	
Natural wood fibers	Motor oil	20	
	Vegetable oil	16	

Sisal	Crude heavy oil	6.4	[46]
Leaves residue		2.7	
Saw dust		6.4	
Coir fiber		5.4	
Sponge gourd		4.6	
Silk-floss fiber		84.6	

Barley straw	1-day weathered oil	11.2	[5]
	7-day weathered oil	12.2	
	Gas oil	7.8	

Wheat Straw	Pure crude oil	2.82	[47]
	Crude oil on freshwater	4.36	
	Crude oil on seawater	4.08	
	Pure diesel	2.77	
	Diesel on freshwatr	3.17	
	Diesel on seawater	2.76	

Bagasse	Heavy crude oil	5.3	[48]
	Light crude oil	3.3	
Rice hull	Heavy crude oil	5.15	
	Light crude oil	3.80	

Banana peel	Gas oil	5.31	[2]
	1day weathered crude oil	6.35	
	7day weathered crude oil	6.63	

Milled PO fibers	Pure crude oil	14	This study
	Oil/water	12.80	
Raw PO fibers	Pure crude oil	5.32	
	Oil/water	4.74	

## Data Availability

The analysis and experimental data used to support the findings of this study are included within the article.
